# Perspectives of Hinduism and Zoroastrianism on abortion: a comparative study between two pro-life ancient sisters

**DOI:** 10.18502/jmehm.v12i9.1340

**Published:** 2019-08-05

**Authors:** Kiarash Aramesh

**Affiliations:** *Assistant Professor, The James F. Drane Bioethics Institute, Edinboro University of Pennsylvania, Edinboro, Pennsylvania, USA; Department of Biology and Health Sciences, College of Science and Health Professions, Edinboro University of Pennsylvania, Edinboro, Pennsylvania, USA.*

**Keywords:** Zoroastrianism, Hinduism, Abortion, Religious bioethics, Pro-life

## Abstract

Hinduism and Zoroastrianism have strong historical bonds and share similar value-systems. As an instance, both of these religions are pro-life. Abortion has been explicitly mentioned in Zoroastrian Holy Scriptures including *Avesta*, *Shayast-Nashayast* and *Arda Viraf Nameh*. According to Zoroastrian moral teachings, abortion is evil for two reasons: killing an innocent and intrinsically good person, and the contamination caused by the dead body (*Nashu*). In Hinduism, the key concepts involving moral deliberations on abortion are *Ahimsa*, *Karma* and reincarnation. Accordingly, abortion deliberately disrupts the process of reincarnation, and killing an innocent human being is not only in contrast with the concept of Ahimsa, but also places a serious karmic burden on its agent. The most noteworthy similarity between Zoroastrianism and Hinduism is their pro-life approach. The concept of *Asha* in Zoroastrianism is like the concept of *Dharma* in Hinduism, referring to a superior law of the universe and the bright path of life for the believers. In terms of differences, Zoroastrianism is a religion boasting a God, a prophet, and a Holy book, while Hinduism lacks all these features. Instead of reincarnation and rebirth, Zoroastrianism, like Abrahamic religions, believes in the afterlife. Also, in contrast with the concept of *Karma,* in Zoroastrianism, * Ahura Mazda* can either punish or forgive sins.

## Introduction

In the history of human civilization, religions have always been major sources of values with huge impacts on the life decisions of their followers. Originating in the dawn of human civilization, Zoroastrianism and Hinduism are two ancient traditions/religions that have adopted a pro-life approach with an emphasis on reverence for life. Although these two sister religions are not compatible in terms of the number of followers (see below), their approaches and perspectives are important and influential in the life decisions of countless people and families around the world.

Abortion is one of the first topics that appeared in the texts and scriptures related to medical ethics from the early days of this field in ancient times, and still is one of the most debated and divisive issues in the field of bioethics. Followers of religions always try to resolve issues such as abortion according to their religion and make their own and their families’ life decisions based on their religious normative approaches.

Zoroastrianism and Hinduism are two ancient inter-related traditions/religions with strong historical bonds that have developed and taken shape in neighboring countries and societies. Studying the similarities and differences between these two religious traditions with regard to an important life-related issue shows the divergent paths of traditions and religions that have the same (or very similar) origins, but have developed in different societies and locations (1).

This paper is the result of a library-based comparative study that has assessed the perspectives of these two religious traditions toward abortion. 

The aim of this paper is to sketch and compare the perspectives of Zoroastrianism and Hinduism on abortion in the light of the unique specifics and characteristics of these two religious traditions, their moral teachings, and their bioethical approaches. For this purpose, these perspectives must be explained by exploring the main sources of Zoroastrian and Hindu bioethics. These sources may either pertain to the theoretical/conceptual teachings of these two religious traditions, or their practical approaches in the real world. By paying attention to the very pro-life nature of these two religious traditions one can clearly see that despite some major differences in the bases of their moral thoughts, both oppose abortion except for certain cases under very distinct conditions.

**Two pro-life traditions and a life issue**

Zoroastrianism and Hinduism both originated among Aryans after their migration to the Middle East and South Asia. Although the theory of the Indo-Aryan migration has also been the subject of scholarly criticism, the similarities and the existence of many common features between the Vedic and Avestan texts indicate a strong ancient interconnection (2). While these two religious traditions had been interconnected before and at the time of the Great Migration, they took separate paths after the settlement of their followers in different geographic areas. Regardless of the causes of this divergence, nowadays there are a lot of differences between these two religious traditions in addition to their original similarities. 

***1.Zoroastrianism***

Zoroastrianism is an ancient Persian religion that was the official religion of the Persian Empire from 600 BCE to 650 CE (3). Estimations on the lifetime of the prophet of this religion, Zoroaster or Zarathustra (Zartosht in current Persian), vary between 8000 and 700 BCE. However, Moubed Dr. Jahangir Ashidari argues that according to historical facts and events, the most realistic estimate of the year of his birth may be 1768 BCE (4).

Zoroaster was born in the present-day Azerbaijan Province in Iran. He moved to Khorasan and the city of Balkh where he declared his prophet hood, and was successful in establishing a new religion. The king of Balkh was among his followers at that time (4).

The most prominent source of Zoroastrian moral thoughts is the religion’s holy book named *Avesta *(5). Only a small part of the current *Avesta* is attributed to Zoroaster himself, as a scripture he brought and left among his people. This part is named *Gatha* and consists of mystical hymns and no concrete jurisprudential or ethical debates (6:155-205). The other parts of *Avesta *are as follow:

- *Yasna*: This is the oldest and most important part of *Avesta*, and includes *Gatha*. It has been argued that this part of *Avesta *has been compiled at the same time as *RigVeda *(see the section on Hinduism below) and there are linguistic similarities between the two (5).
- *Yashtha*: This part of Avesta is mostly poetic and includes verses of worship to *Ahura Mazda* and *Amshaspandan* (see below). *Yashtha* consists of poems and epics, and does not include moral or jurisprudential elements or teachings (5).
- * Visparad*: *Visparad* means lords and leaders. This part of *Avesta* includes cosmological and ontological teachings. It also contains general moral wisdom for people, describing the best behavioral models for men and women (6).
- * Vandidad*: This is the jurisprudential part of *Avesta*. It was compiled centuries after the death of Zoroaster and mostly explains how Zoroastrian clergy thought or acted in issuing jurisprudential decrees. *Vandidad* is partly related to medical issues such as abortion (5) (see below).
- *Khordeh Avesta*: In 400 CE, Moubed Azarbad MehrAspand compiled this part of *Avesta* to teach Zoroastrian rituals to people. At that time, Zoroastrianism was the official religion of the Sassanids, who were the last dynasty before Islam and ruled over the Persian Empire for more than 200 years (5).

In addition to the *Vandidad* part of *Avesta*, there are other holy scriptures like *Arda Viraf Nameh* and *Shayast-Nashayast* that are rich in ethical and jurisprudential teachings. These have been compiled in the centuries after the lifetime of Zoroaster, mostly during the dominance and prevalence of Zoroastrianism in the Persian Empire, from about the 5^th^ century BCE to the 7^th^ century CE (7).

Through the seventh and eighth centuries CE, Persia gradually joined the Muslim world and the dominance of Zoroastrianism ended. Nevertheless, the cultural influence of this religion has persisted until contemporary times (8). Nowadays, the followers of Zoroastrianism mostly live in Iran, India (the Parsis) and Western countries. Estimations of the present population of Zoroastrians worldwide differ between 145,000 and 2.6 million (9). Beyond the community of its formal believers, the current and historical influences of Zoroastrianism on the Iranian culture and even the Iranian version of Shiite Islam have been significant. It has been argued that the Iranian/Persian culture is a mixture of three different heritages: The Islamic/Shiite religion/culture, the ancient Persian/Zoroastrian culture, and the impact of the Western/modern culture in recent centuries (10).

Some foundational features of Zoroastrianism that are very important in understanding the spirit of this religion and its bioethical perspectives are as follow: 

*Monism vs. Dualism*

Zoroastrianism is a monotheistic religion. The dualism of *Ahura Mazda* and *Ahriman* in the Zoroastrian cosmology has been translated into a dualistic view in theological and moral perspectives (4). Therefore, Zoroastrian morality is largely based on a type of dualism that believes in the timeless and everlasting combat between good (*Ahura Mazda/Sepand Minu/Ashuns*) and evil (*Ahriman/Angra Minu/Doruj*). It is noteworthy that Zoroastrianism in its dualistic moral view is more similar to Abrahamic religions than to Hinduism and other Asian religions (4).

According to the Zoroastrian dualistic view, *Ahura Mazda* created all the good in the universe, and *Ahriman* created all the evil (8). Human beings were also the creation of *Ahura Mazda*, and are therefore considered intrinsically good. However, they have the ability and autonomy to choose between good, which is in concordance with their nature, and evil, which is suggested and encouraged by *Ahriman*. The former follows *Asha* as the divine rule of existence and are called the *Ashuns*, while the latter who choose evil (*Doruj*) are named the *Dorvands* (followers of *Doruj*/evil/lie) (11).

According to the aforementioned beliefs and perspective, which consider every unborn human being as a creature of and a future soldier for *Ahura Mazda*, Zoroastrianism is a pro-life religion. Some of the newer parts of *Avesta* explain punishments and difficult steps for purgation of a person who has committed abortion (7).

*Amshaspandan and Asha*

Before the time of Zoroaster, the Aryans, including the group that moved to India and are called Hindus, used to worship multiple gods and goddesses. Zoroaster introduced a single God named *Ahura Mazda*, and the previous Aryan gods were then revived as the various reflections or faculties of that single God; these were named the *Amshaspandan*, and were inseparable from *Ahura Mazda. Amshaspand* means “the immortal pure” and *Amshaspandan *is the plural form of *Amshaspand. * This word is constituted of two parts: *Amesha* and *Sepanta*. *Amesha* means immortal and indestructible, and it also specifies everlasting and beneficent entities such as the four elements, the sun, and *Houm* (healing plant). *Sepanta* means generous, merciful, creator and pure (4).

According to Zoroastrian teachings, the *Amshaspandan *are as follow:

- *Asha*: This is a very important concept in Zoroastrianism and is rather similar to the concept of *Dharma *in Hinduism (4). *Asha* means the eternal law, righteousness, and the unchanging rules of the universe and humanity. People who follow *Asha* and believe in it as the divine rule of existence are the *Ashuns*, while others who choose evil (*Doruj*) are the *Dorvands* (11).
- *Vahumana*: Good behavior, character, and intent.
- *Xashtra*: God’s city, God’s power, and God’s faculty.
- *Armeity*: Love, devotion, and purity.
- *Heorutat*: Growth, Development, and Happiness.
- *Amortat*: Immortality, and indestructibility (4).
- *Nashu*: Being clean and pure is very important in Zoroastrian teachings and rituals (3).* Nashu* is uncleanliness or a demon, mainly attributed to dead bodies (3). Any person contaminated with *Nashu* should be cleaned through a set of sophisticated rituals including being washed with a liquid prepared from cow’s urine (3). Zoroastrians do not bury the bodies of the dead because they believe that this practice contaminates the soil. Instead, they leave corpses in places named *dakhma* to be eaten by wild animals and degraded by natural forces (3). Since an aborted fetus is a dead body, abortion is considered to contaminate the mother’s body with *nashu, which* is a great sin (see below for further discussion) (7).

***2.Hinduism***

Claimed to be the oldest living religion in the world, Hinduism is a huge network of concepts, beliefs and rituals initiated more than two thousand years ago in ancient India. Today, Hinduism has about 900 million followers all around the world. Most Hindus live in India and Nepal, but they also shape large populations in other Asian countries like Cambodia, Thailand, Burma and Indonesia. In addition, in developed countries like the United States and the United Kingdom, Hindus are among sizeable minorities.

Spiritual teachings of Hinduism and its sages and spiritual masters have had a great influence on Western cultures over the recent decades. Hindu spirituality in many direct and indirect forms has changed the culture, spirituality and lifestyle in Western societies. As an example, one can mention Yoga, which originated in Hindu traditions, and has become very popular in Western countries in the past century.

It is interesting to explore the origin of the word “Hindu”. As a huge cultural network, Hinduism was born in ancient India, but the name “Hindu” was acquired in the medieval centuries to differentiate the religion from others such as Islam (12). As a matter of fact, the word “Hindu” comes from Persian literature. Persian geographers coined the name “Hindu” for people who lived beyond the river Indus (Sindhu) (13). Addition of the suffix “-ism” is a legacy of British colonialism in the 19^th^ century. 

In ancient India, Hinduism was traditionally called “*Sanatana Dharma*”, which connotes the most central concept in this tradition, but cannot be fully translated into English. However, some have chosen “eternal law” as an equivalent.

It is difficult, if not impossible, to try to find a set of essentials for all the sects, groups and denominations within the circle of Hinduism. One cannot specify a concept, belief, ritual or other element as the common - or defining - feature of this religion. In fact, features like reverence for Vedas (the ancient Scripture of Hinduism), believing in a system of values named* Dharma*, and even belonging to the Indian nation have been mentioned as unifying features of Hinduism, but none is common among all Hindus.

Therefore, Hinduism can be understood as a network of inter-related ideas without a single unifying feature. In fact, instead of one or a few essential common and all-embracing features, one can speak about a wide network with a series of overlapping similarities reminiscent of “family resemblance” as explicated by Ludwig Wittgenstein for defining other phenomena such as art (14).

Some scholars argue, however, that the concept of family resemblance cannot solve the problem of lack of common features in the search for Hindu moral principles. Although the above-mentioned “family resemblance” means that no single unifying essential feature can be found for Hinduism, some major characteristics can be identified, which are 1) common among most sects and branches of Hinduism, and 2) essential and representative of the nature and main directions, teachings, key concepts, and values of this tradition. A non-inclusive list of these characteristics is presented below.

- *Unity in the Midst of Plurality*

One characteristic of Hinduism is the existence of numerous forms of supreme beings, as can be seen in the enormous number of deities. *Shiva*, *Shakti*, *Vishnu*, *Ganapati*, *Surya*, and *Subrahmanya* are the deities worshiped by different sects of Hinduism, but can be considered as different manifestations of a single supreme being. This interpretation of the Hindu tradition, which makes it similar to monotheistic religions, is compatible with a famous verse of *Rigveda*: “Reality is one; sages call it by different names”; or this verse of *Bhagvad Gita*: “Even those who are devoted to other gods and worship them in full faith, even they, O *Kaunteya*, worship none but Me” .

This plurality is not confined to the deities. For instance, Hinduism does not have a single founder, but seems to have been created and formed by accumulation of teachings and revelations of numerous sages, gurus and spiritual masters in ancient India (15).

This characteristic provides Hinduism with an inimitable flexibility and respect for plurality and diversity, which (alongside other qualities like the central concept of non-violence, *Ahimsa*) were very important in the history of this religion and that of India. For example, one can mention the historical acceptance of Jewish and Zoroastrian immigrants whose lands had been invaded by Romans and Muslim Arabs respectively. Another case in point is the specifics of the democracy founded by Mahatma Gandhi in this huge subcontinent with such a unique variety in cultures, religions, and ways of life.

- *The Concept of Dharma *

*Dharma* holds the human community and the entire world together. As explained above, this concept is very similar to the concept of *Asha* in Zoroastrianism. In Hinduism, *Dharma* illuminates humans’ responsibilities and way of life. As mentioned above, in the ancient Indian subcontinent, the followers of Hinduism called their religion/tradition *Sanatana Dharma *in which the word *Sanatana* means eternal (15). Also, in Zoroastrianism, the people who are true followers of Zoroaster are called *Ashun. *Therefore, it seems that attributing followers to the eternal law is a common concept in both Zoroastrianism and Hinduism.

- *Concepts of Karma, Samsara, and Reincarnation*

Karma is one of the most important concepts in Hindu ethics and morality. This concept denotes that a law of cause and effect rules the world of human deeds, both mentally and physically. Each action produces its own reaction in the world. Accordingly, a good action has a good reaction for the human agent in his/her current life or next lives, while a bad action will certainly bring about bad consequences, which, again, can take place in the current or subsequent lives of the human agent. This continuous cycle of action, reaction, birth, death and rebirth is called *Samsara*. This cycle is not endless. One can break the cycle of *Samsara* by good deeds that lead to salvation and getting out of the cycle. This salvation, called *Muksha* (or *Nirvana* in Buddhism and Jainism), is the ultimate goal of life. Therefore, the final purpose of Hindu ethics is salvation that is manifested in breaking the cycle of *Samsara* and entering the eternal salvation, sometimes named *Muksha *(15)*.*

There are serious controversies among scholars on the existence of a Hindu Bioethics. Like other ancient civilizations, the Indian subcontinent had its own medicine and healing tradition called *Ayurveda* (the science of life), which was a sort of humoral medicine (16). The existence of this medicine and its rich literature, mixed with Hindu teachings and thoughts about humanity and morality, led some scholars to try to derive from it a kind of Hindu biomedical ethics. For example, the ancient Hindu stories about gods with human bodies and animal heads were used to conclude the permissibility of Xenotransplantation in Hindu bioethics (13).

Some scholars, however, do not agree with this method of constructing Hindu bioethics (17). They argue that the mere existence of these traditional schools of medicine in the mostly Hindu ancient Indian subcontinent does not imply that their literature mirrors Hindu bioethics (13).

The key point in this regard is that there is no consensus among Hindus on all of the concepts and principles attributed to this religion. This vast diversity, as mentioned above, is one of the most important characteristics of Hinduism. This characteristic reflects itself in Hindu ethics, applied ethics, and bioethics (13).

The main question is, how can all these sects and branches of Hinduism agree upon a set of principles for applied ethics, since they cover such a diverse variety of beliefs but have no common feature (such as a prophet or a holy book, as is the case with Christianity, Islam, or Buddhism)? Therefore, the existence of a Hindu bioethics with a distinct set of principles has been a subject of controversy and debate. Two kinds of efforts, however, have been made to solve this problem:

1. Some scholars have pointed out common concepts, like *Karma*, as the core and unifying concept of Hinduism and Hindu ethics. By doing so, however, they have broadened the scope of Hinduism in a way that even Buddhism and Jainism can be considered some sort of Hinduism. It is obvious that this is too wide-ranging to serve the purpose (17).

2. Some other scholars have tried to choose just one sect or group within the wide spectrum of Hinduism, and described Hindu ethics based only on the values and beliefs of that sect or group. They have been successful in finding a set of principles, but the results cannot be called “Hindu Bioethics” as they are too narrow in range (13).

The aforementioned endeavors, however, show a very historically obvious fact: that the impossibility of attributing a set of common and all-encompassing principles and values to Hindu morality and applied ethics does not mean it is impossible to speak about Hindu bioethics. Three main categories of sources can be used to delineate the content of Hindu bioethics, including its values, principles, teachings, and judgments. These categories are as follow:

1- Every system or set of values, moral principles and ethical deliberations that finds its roots in the Hindu religion/tradition can be considered and named Hindu ethics, regardless of how many Hindu sects and groups it is shared among. When it comes to value-judgments about medicine, healthcare and life sciences, these principles definitely shape Hindu bioethics. By the same token, we can reach a set of principles, concepts and values that are not all-encompassing and unifying, but still characterize this very brand of religious bioethics.

2- *Ayurveda* and other branches of Indian traditional medicine have been used as a rich source of Hindu reflections on Human life, death, suffering and so on. Ayurvedic classical texts like *Caraka Samhita* and *Sustuta Samhita *are among the sources of Hindu reflections about human body and self that have major implications for bioethics (16).

3- Deliberations and reflections of Hindu scholars on different sorts of bioethical issues provide another main source for delineation Hindu bioethics. Hindu scholars, sages and spiritual masters have discussed issues like abortion, futile treatment, organ transplantation, contraception and mercy killing. What they have written, taught or told are a rich source for studying Hindu bioethics. Also, one can induct methods of Hindu bioethics by observing the ways in which Hindus have approached the above issues and reached judgments and conclusions about them.

In their bioethical deliberations, Hindu scholars appeal to Hindu concepts like *Karma*, *Dharma* (as described above), *Ahimsa *(non-violence) and respect for life and nature. They also appeal to classic texts and scriptures of the religion/tradition from the oldest existing ones, namely Vedas, to other essential ones like *Upanishadha* or *Bhagvad Gita*. One example of such references to classical scripture is described above on the issue of Xenotransplantation (17).

Hindu Bioethics should be seen as a lived experience**. **From ancient “Vedic healers” to modern healthcare professionals, numerous generations of physicians and clinical practitioners in the Indian subcontinent have sought the values and principles governing their practice in one of the oldest and richest religions and traditions in the world, that is, Hinduism. The spirit of the subcontinent shaped and determined the nature of this value system throughout its long history. This Indian spirit is what gives the Hindu bioethics a sort of unity in the midst of such vast and wide diversity.

Hinduism has its own perspective on fundamental aspects of human life. According to this perspective, the moral energy is preserved in the form of *Karma*, and death is not the opposite of life, but is the opposite of birth. This characteristic makes Hinduism different from Abrahamic religions in which the will of God determines the consequences of good or bad deeds, rather than a natural rule like Karma (14). In Hinduism, the ultimate purpose of human beings is liberation from the circle of birth, death and rebirth, instead of entering heaven as is the case in Abrahamic religions (15).

Obviously, none of the aforementioned features is unique to and common among all the sects of Hinduism. Altogether, however, these features are the different surfaces of an underlying spirit: the spirit of Hinduism, which is the spirit of the Indian subcontinent. This spirit has been the source of inspiration for successive generations of sages, gurus and spiritual masters.

The reverence for life and a strong tradition of non-violence (*Ahimsa*) has shaped the perspectives of Hindu bioethicists towards key bioethical issues like abortion, euthanasia and brain death (15).

Virtue ethics also exists in some Hindu ethical teachings. This approach to ethics focuses mainly on the moral agent instead of the act itself or its consequences. Accordingly, going through a process of self-purification results in achieving a moral character that always chooses to perform the ethically right deeds (18). 

At the end, the practical results of this type of virtue ethics are somehow different from those of its counterparts in the West or the Middle East. This difference is rooted in the spirit of Hinduism and the Indian subcontinent, and has a great impact on the moral character of the virtuous person.

In sum, one can conclude that despite the diversity, which is one of the main characteristics of Hinduism, it is possible to delineate some major concepts that shape the infrastructures of morality in this religion/tradition. In the same way, one can sketch the principal values and directions of Hindu bioethics. In addition, the present study has pointed out three main sources for bioethical endeavors within the Hindu tradition/religion:

- Value-judgments and moral deliberations rooted in and performed within the Hindu tradition
- Textbooks and the heritage of ancient Hindu medicine, including Ayurveda
- Reflections and deliberations made by Hindu scholars on bioethical issues that have accumulated throughout a long history, including the modern era

Hindu bioethics can be sought and learned as the collective lived experiences of Hindus on traditional and modern issues that are of biomedical nature. These experiences, which have been accumulated collectively throughout the Indian subcontinent and have produced a huge body of literature, are the very nature and unifying umbrella that cover a long history of ethical and moral endeavors of a vast array of sects, branches and groups within the old religion/tradition of Hinduism.

 **The importance of the issue of abortion **

Abortion is the intentional termination of the life of an unborn human embryo or fetus. This act is forbidden and considered as inherently evil in all major religious traditions of the world. In the modern era, however, the situation has changed. Many factors contributed to bringing abortion to the top tier of the most heated ethical debates among the general public and scholars, and making some moral and religious thinkers and authorities rethink and reconsider the absolute evilness of abortion, at least its indirect forms. The issue of population growth in a number of societies has caused some policy-makers to see abortion as a means for population control and prevention of unwanted and unplanned births.

The largest Hindu population in the world lives in the Indian subcontinent, the birthplace of Hinduism (12). In addition, Hinduism reflects the very spirit of the subcontinent. Therefore, when speaking about abortion in Hinduism, it is important to take a look at the realities of its geographical setting. According to the Indian law, abortion is permitted until the twentieth week of pregnancy, only for medical and a very limited number of social reasons.

One of the social reasons for a massive number of abortions in India is the gender of the fetus. When prenatal sex determination by ultrasound became available, many families killed their unborn daughters to get rid of the social and economic burdens of having a daughter and sometimes hoping to have baby boys in the next possible pregnancies.

The selective abortion of female fetuses has increased in India over the past few decades. The 2011 census showed 7.1 million fewer girls than boys aged younger than seven, which showed an increase compared to the 6 million in 2001 and 4.2 million in 1991. The sex ratio in this age group is now 915 girls to 1,000 boys, the lowest since such records began to appear in India in 1961. Parents have little problem with their first child being a girl, but want their second to be a boy. In these families, the gender ratio for second births has fallen from 906 girls per 1,000 boys in 1990 to 836 in 2005, implying that an estimated 3.1 to 6 million female fetuses have been aborted in the past decade. It has even been claimed that approximately eight million female fetuses may have been aborted in the past decade, which has been called a “national shame”.

**Similarities**

Abortion has been explicitly mentioned in the Zoroastrian Holy Scriptures including *Avesta*, *Shayast-Nashayast* and *Arda Viraf Nameh*. In addition to regarding abortion as evil and forbidding it, these books prescribe some brutal punishments for women who commit abortion in the afterlife (7).

In addition to condemning abortion in the Holy Scriptures, Zoroastrianism provides moral reasoning, according to its own system of beliefs, for regarding abortion as evil. According to the Zoroastrian moral teachings, abortion is evil for two reasons: killing an innocent and intrinsically good person, and the contamination caused by the dead body (*Nashu*) (7).

On the other hand, as described above, the main sources of Hindu bioethics, which are its concepts and traditions, shape its approaches to ethical issues at the margins of life, including abortion. When it comes to the abortion debate, the principal concepts involving moral deliberations are *Ahimsa*, *Karma*, and reincarnation. Accordingly, abortion deliberately disrupts the process of reincarnation and kills an innocent human being; therefore, it is in contrast with the concept of *Ahimsa *and imposes serious karmic burdens on its agent. In addition, in major resources of Hinduism, abortion has been strongly condemned, which confirms the pro-life approach of this religion/tradition towards abortion. According to Hindu bioethics, abortion is allowed only in cases where it is necessary for saving the life of the mother. The perspective of Hinduism is a very pro-life one, emphasizing Ahimsa and its intrinsic reverence for life. 

It should be mentioned that in addition to the similarities explained below, there are others in minor aspects such as rituals. For example, considering the cow as a sacred animal and using its urine for cleaning the body after abortion is common practice in both traditions/religions.

***Dharma vs. Asha***

The concept of *Asha* in Zoroastrianism is similar to the concept of *Dharma* in Hinduism. Both *Asha* and *Dharma* refer to a superior law of the universe and the bright path of life, which should be adopted by the believers. 

In the Indian subcontinent, before their historical encounter with other religions and traditions, the followers of Hinduism called their religion/tradition *Sanatana Dharma. *The word *Sanatana* means eternal (15). Also, in Zoroastrianism, the true followers of Zoroaster are called *Ashun. *Therefore, it seems that attributing followers to the eternal law is a common concept between Zoroastrianism and Hinduism.

The approaches of these two religions to moral issues like abortion are consistent with this ontological view of the universe. The entire universe is created and ruled in accordance with Dharma/Asha, and all the people should follow these eternal rules. Morality ultimately means consistency and accordance with these higher entities. In both religions, abortion is a violation of the higher and sacred law of the Universe and existence. Therefore, abortion, like murder, robbery and other kinds of immoral behaviors, is wrong and unacceptable.

***Reverence for life***

The most noteworthy similarity between Zoroastrianism and Hinduism is their pro-life approaches. In both religions/traditions, abortion is considered murder and is forbidden.

Ayurveda and other branches of Indian traditional medicine have been used as a rich source of Hindu reflections on Human life, death, suffering and so on (15). Deliberations and reflections of Hindu scholars on different sorts of bioethical issues provide another main source for delineating Hindu bioethics. 

In their bioethical deliberations, Hindu scholars appeal to Hindu concepts like *Karma*, *Dharma* (as described above), *Ahimsa *(non-violence) and respect for life and nature. They also appeal to the classic texts and scriptures of the religion/tradition from the oldest existing ones, namely Vedas, to other essential ones such as *Upanishadha* or *Bhagvad Gita*^.^ (19)

Abortion is mentioned in early Vedic scriptures. For example, in *Brahmanas*, the second major body of Vedic literature, abortion is considered a crime (19: 22-23), and the same approach is adopted by *Upanishads *(19)*.* Other classical scriptures of Hinduism have also expressed their opposition to abortion in several ways, for instance by comparing abortion with killing a priest, considering abortion a sin worse than killing one’s parents, and threatening the mother to lose her caste. 

In the modern world, Hindu sages and scholars have continued to condemn abortion. As Mahatma Gandhi once wrote, "It seems to me clear as daylight that abortion is a crime.” It can be argued that the traditional concepts of reverence for life and non-violence (*Ahimsa*) have been most influential on the perspectives of Hindu bioethicists towards key bioethical issues such as abortion, euthanasia and brain death (15). As explained above in this paper, *Ahimsa* is a core concept in the approach of Hinduism to the issue of abortion. As mentioned above, Ahimsa is based on the sacredness of all creatures as manifestations of the Supreme Being. 

The reverence and love granted to all manifestations of life results from the very concept of *Ahimsa*, which has made the Hindu religion/tradition a strongly prolife one. This pro-life attitude has found its way from Hinduism to other Asian religious traditions (18, 20). 

In Zoroastrianism, abortion is regarded as killing an innocent and intrinsically good person. Concepts like *Ahimsa* do not exist in Zoroastrianism, but reverence for human life does. As explained above, morality in Zoroastrianism is based on a polarized account of the Universe as the everlasting battleground of good and evil, that is, *Ahura Mazda* and *Ahriman *(4). Since the human being is intrinsically good and has been created by *Ahura Mazda*, killing an unborn embryo or fetus is a violation against the forces of *Ahura Mazda* and a contribution to the forces of *Ahriman.* Therefore, abortion is considered a major sin. Accordingly, it is not surprising that he Holy Scripture of Zoroastrianism equates abortion with murder and rules punishments for persons who commit it. Also, in other parts of *Avesta*, there are revelations describing brutal punishments for such people in the afterlife (7).

***Exceptions for the ban***

When it comes to abortion, in addition to adopting a pro-life approach, both religions recognize some exceptions for their ban on abortion. In both traditions/religions abortion is permitted when the life of the mother is in danger. Therefore, both give priority to the mother’s life over the life of her unborn child.

As a matter of fact, although both Zoroastrianism and Hinduism ban abortion except for cases in which mothers’ lives are endangered, the bioethical bases of this ban in these two religions are different from each other. In Zoroastrianism, the ban is based on abortion being the same as killing an innocent person, and the contamination caused by the dead body. But in Hinduism, it is based on the law of *Karma* and depriving a person from one cycle of his or her rebirth. However, regardless of the theoretical bases and theological justifications, both religions give priority to the lives of the mothers over the lives of their unborn children.

The recognized exceptions raise a question about the moral status and personhood of the embryo. Although not mentioned directly in the original manuscripts, it seems that both these religions regard a moral status for the human embryo from the very first stages of life. This attitude is similar to the perspective of the Catholic Church that believes in recognition of personhood from the time of conception. However, a minority of Hindus believe that incarnation takes place in the 7^th^ month of pregnancy (21). Also, it has been shown that the majority of Zoroastrians are not against sperm and egg donation that necessitates in Vitro Fertilization (22). This position makes Zoroastrianism different from classical Catholicism or other recent pro-life movements (23). 

**Differences**

A comparative study will not be complete without describing the differences between the subjects of comparison. Although Zoroastrianism and Hinduism are ancient sister religions that originated among the same group of people (Aryans) after the Great Migration, their followers settled in two different neighbor countries: Persia and India. Living in separate contexts and conditions naturally has had its consequences. As mentioned above, Zoroastrianism is more similar to Abrahamic religions than to *Dharmic* ones in many ways. The main differences between these two religious traditions in terms of their perspectives on abortion are described below.

***Unity vs. diversity***

One of the main differences between Zoroastrianism and Hinduism is in the very fact that Zoroastrianism is a religion with a God, a prophet, a Holy book, and in long periods of its history, a single hierarchical order of clergies. Hinduism, however, lacks all these features. There is no single god, prophet, holy book or system of clergies shared among all the groups, sects and communities who call themselves Hindu. Therefore, in order to find the normative positions of Zoroastrianism, for example their perspective on abortion, one can rely on a single defined set of resources. In Hinduism, however, each expressed viewpoint only belongs to a number of believers and does not reflect the viewpoint of all religions/traditions. Considering this difference between these two religions is important for reading and understanding all the scholarly works that have been published in this regard.

In other words, Zoroastrianism is a typical religion, while Hinduism is a mixture of similar and interrelated traditions/religions. However, considering the familiar resemblance that ties the members of this group to each other, one can consider Hinduism a unique, vast tradition reflecting the spirit of the Indian subcontinent.

***Afterlife vs. reincarnation***

One of the most important differences pertains to the concepts of rebirth and reincarnation. Unlike Hinduism, Zoroastrianism does not believe in reincarnation and rebirth, but believes in the afterlife, like Abrahamic religions.

Therefore, in Zoroastrianism, abortion is not considered as depriving a person of a cycle of human life, but as denying him or her the only chance of birth and enjoying life on earth.

***Karma vs. omnipotent God***

In Hinduism, killing a living creature, including a fetus, is regarded as interfering in its spiritual evolution. Such interference places Karmic burdens on its agent. Therefore, according to the natural law of *Karma*, the agent(s) of such a crime will definitely encounter it’s just punishment/retaliation in their current or next lives. 

As an example of how the concept of *Karma *works with regard to abortion, it has been said that abortion is a kind of punishment for meat-eaters. The fetus was a meat eater in his or her previous life, while the mother was a cow in her previous life, now taking revenge according to the rules of nature. According to this belief, meat-eaters and other people who kill live entities cannot escape the retribution set by the laws of *Karma*; thus, in their next lives, they will have to undergo the misfortune, and may be recurrently aborted.

It is obvious that the karmic maleficence of abortion is in close relation to reincarnation. This very belief that a human embryo is essentially a human person underlines the karmic effect attributed to abortion in Hinduism (19). The concept of *Caraka* (*Caraka*’s theory of causality) shows how the karmic burden/heritage of past lives is transferred to the unborn fetus (19). Therefore, killing the unborn child disrupts this process of transferring the *Karma* and imposes karmic burdens on its agent who, by his/her act of abortion, has deprived the unborn baby of one of his or her chances to pursue salvation in a human life.

Cleanliness, on the other hand, is a very major concept and an emphasized duty for believers in Zoroastrianism. One of the most offensive contaminants that can affect the cleanliness of the human body is a corpse. Accordingly, there are specific burial rituals in Zoroastrianism to prevent contamination of the soil, fire and living bodies by a corpse. According to Zoroastrian teachings, abortion exposes the body of the mother to contamination caused by the dead body of the aborted fetus. Therefore, in addition to abortion being forbidden, there is a multi-step ritual for purgation of the body of the mother, including washing her womb with a liquid made from cow urine (7).

The concept of *Karma*, as it exists in Hinduism, has no place in Zoroastrianism. Based on Zoroastrian teachings, *Ahura Mazda* can punish or forgive sins. Therefore, the punishment or forgiveness of bad deeds do not occur as a result of a natural law, but is attributed to *Ahura Mazda*, who can either punish or forgive the sinner (4). As a matter of fact, belief in an omnipotent God is not consistent with the concept of *Karma*, because accepting the inviolability of this concept as a natural law ties the hands of God. 

In Zoroastrianism, like Abrahamic religions, the omnipotent God defines what is good and what is evil, and punishes or forgives anyone He wants. Therefore, He is the one who can establish the immorality of abortion and offer punishment or forgiveness.

## Conclusion

Zoroastrianism and Hinduism are similar to each other in adopting strong pro-life approaches to issues like abortion. Although with different theoretical bases, both these religious traditions ban abortion and allow it only if the life of the mother is threatened by continuation of the pregnancy. Also, they are fundamentally different in the conceptual and theological bases of their moral approaches. 

Zoroastrianism provides moral reasoning for regarding abortion as evil according to its own system of beliefs. On the other hand, in Hindu bioethics, the principal concepts involving moral deliberations on abortion are *Ahimsa*, *Karma*, and reincarnation. Accordingly, abortion as deliberately disrupting the process of reincarnation and killing an innocent human being is in contrast with the concept of *Ahimsa* and brings serious karmic consequences for its agent. Hindu bioethics condemns abortion and allows it only in cases where abortion is necessary for saving the life of the mother.

The concept of *Asha* in Zoroastrianism is similar to the concept of *Dharma* in Hinduism. Both these concepts refer to a superior law of the universe and the bright path of life. The most noteworthy similarity between Zoroastrianism and Hinduism, however, is their pro-life approach. 

The perspectives of Hindu bioethicists on key bioethical issues such as abortion has been shaped by a strong tradition of non-violence (*Ahimsa*) and an immense reverence for life. *Ahimsa* is a core concept in the approach of Hinduism to the issue of abortion and is based on the sacredness of all creatures as manifestations of the Supreme Being. In Zoroastrianism, abortion is regarded as killing an innocent and intrinsically good person. Since the human being is essentially good and has been created by *Ahura Mazda*, killing an unborn child is a violation against the forces of *Ahura Mazda* and a facilitator to the forces of *Ahriman*, hence a major sin. Therefore, both these religions have adopted a pro-life approach toward the abortion debate.

In both traditions/religions abortion is permitted when the life of the mother is in danger. Therefore, both give priority to the mother’s life over the life of her unborn child.

One of the main differences between Zoroastrianism and Hinduism is in the fact that Zoroastrianism is a religion with a God, a prophet, a Holy book, and in long periods of its history a single hierarchical order of clergies. Hinduism, however, lacks all these features. 

Another important difference between Zoroastrianism and Hinduism is related to the concepts of rebirth and reincarnation. Like Abrahamic religions, Zoroastrianism believes in the afterlife. Therefore, in Zoroastrianism, abortion is not considered as depriving a person of a cycle of human life, but it is considered as depriving a person of his or her only chance to be born and enjoy life on earth.

In Hinduism, killing a living creature, including a fetus, is regarded as interfering in its spiritual evolution, and places Karmic burdens on its agent. Therefore, according to the natural law of *Karma*, the agent(s) of such a crime will definitely encounter just punishment/retaliation in their current or next lives. 

The concept of *Karma*, as advocated by Hinduism, has no place in Zoroastrianism. In Zoroastrianism, punishment and forgiveness of bad deeds are not the result of a natural law, but are administered by *Ahura Mazda*, who can either punish or forgive the sinner.

In sum, one can conclude that Zoroastrianism is similar to Abrahamic religions in its approach to abortion, and this is what makes it different from its *Dharmic* sister, Hinduism. Although both these ancient sister religions have adopted pro-life approaches, they are very different in many aspects and features. Analyzing the historical course and reasons for the emergence of these differences can be a subject for further studies in the future.

## Citation to this article:

Aramesh K. Perspectives of Hinduism and Zoroastrianism on abortion: a comparative study between two pro-life ancient sisters. J Med Ethics Hist. 2019; 12: 9.
